# A triclinic polymorph of *catena*-poly[[bis­(*N*,*N*-dimethyl­formamide-κ*O*)cobalt(II)]-di-μ-1,5-dicyanamido-κ^4^ *N* ^1^:*N* ^5^]

**DOI:** 10.1107/S1600536812043310

**Published:** 2012-10-24

**Authors:** S. C. Meng

**Affiliations:** a School of Chemistry and Chemical Engineering, Jiangsu University, Zhenjiang 212013, People’s Republic of China

## Abstract

The title compound, [Co(C_2_N_3_)_2_(C_3_H_7_NO)_2_]_*n*_, is a triclinic polymorph of the previously reported monoclinic structure [Tong *et al.* (2003 ▶). *Acta Cryst.* E**59**, m405–m407]. The Co^II^ ion lies on an inversion centre and adopts an almost regular octa­hedral N_4_O_2_ coordination geometry. Adjacent Co^II^ atoms are connected by two bridging dicyanamide ligands, resulting in the formation of neutral chains parallel to the *b* axis. The title complex is isotypic with the Mn^II^ analogue but not with the Ni^II^ analogue.

## Related literature

For the design and synthesis of metal-organic compounds, see: Long & Yaghi (2009 ▶). For the structures of the Mn^II^ and Ni^II^ analogues, see: Batten *et al.* (1999 ▶); Shen & Yuan (2005 ▶). For the structure of the monoclinic polymorph, see: Tong *et al.* (2003 ▶).
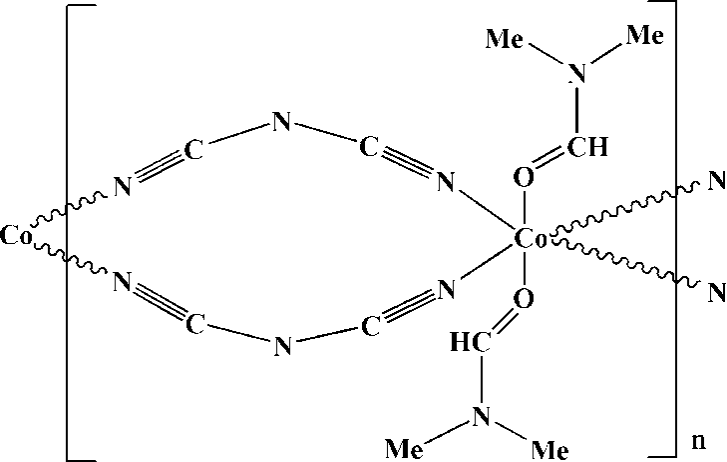


## Experimental

### 

#### Crystal data

[Co(C_2_N_3_)_2_(C_3_H_7_NO)_2_]
*M* *_r_* = 337.22
Triclinic, 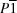

*a* = 6.4315 (13) Å
*b* = 7.3879 (15) Å
*c* = 8.6210 (17) Å
α = 105.69 (3)°
β = 107.94 (3)°
γ = 96.19 (3)°
*V* = 366.93 (17) Å^3^
*Z* = 1
Mo *K*α radiation
μ = 1.19 mm^−1^
*T* = 150 K
0.22 × 0.18 × 0.15 mm

#### Data collection

Rigaku Saturn724+ diffractometer
Absorption correction: multi-scan (*CrystalClear*; Rigaku, 2008 ▶) *T*_min_ = 0.845, *T*_max_ = 1.000
2514 measured reflections
1319 independent reflections
1242 reflections with *I* > 2σ(*I*)
*R*_int_ = 0.015

#### Refinement

*R*[*F* ^2^ > 2σ(*F* ^2^)] = 0.025
*wR*(*F* ^2^) = 0.061
*S* = 1.04
1319 reflections
97 parameters
H-atom parameters constrained
Δρ_max_ = 0.22 e Å^−3^
Δρ_min_ = −0.24 e Å^−3^

### 

Data collection: *CrystalClear* (Rigaku, 2008 ▶); cell refinement: *CrystalClear*; data reduction: *CrystalClear*; program(s) used to solve structure: *SHELXTL* (Sheldrick, 2008 ▶); program(s) used to refine structure: *SHELXTL*; molecular graphics: *SHELXTL*; software used to prepare material for publication: *SHELXTL*.

## Supplementary Material

Crystal structure: contains datablock(s) I, global. DOI: 10.1107/S1600536812043310/rz5015sup1.cif

Structure factors: contains datablock(s) I. DOI: 10.1107/S1600536812043310/rz5015Isup2.hkl

Additional supplementary materials:  crystallographic information; 3D view; checkCIF report

## Figures and Tables

**Fig. 1.**
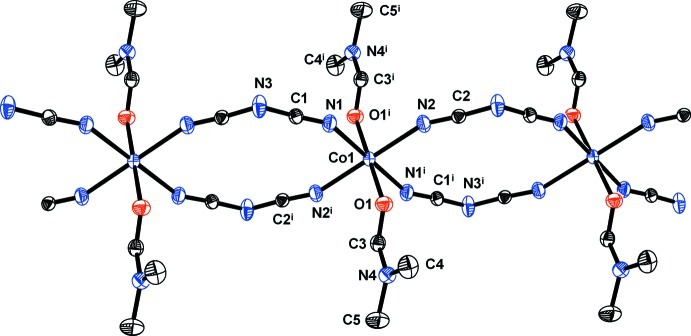
The polymeric structure of the title compound, with atom labels and 30% probability displacement ellipsoids. All H atoms have been omitted. Symmetry code: (i) 1 - x, -y, 1 - z.

## supplementary crystallographic information

### Comment

The design and synthesis of metal-organic compounds have attracted great attention in recent years (Long & Yaghi, 2009), in particular focusing on the properties of flexible bridging ligands able to construct metal-organic compounds with various structures. The title compound is constructed by the flexible dicyanamide bridging ligand through diffusion reaction.

As illustrated in Fig. 1, the cobalt(II) ion lies on an inversion centre and adopts an octahedral coordination geometry. Metal atoms are connected by two dicyanamide bridging ligands, resulting in the formation of neutral chains parallel the *b* axis. The title complex is isotypic with the Mn analogue (Batten *et al.*, 1999) but not with the Ni analogue (Shen & Yuan, 2005). A monoclinic polymorph of the title compound was previously reported (Tong *et al.*, 2003).

### Experimental

Co(NO_3_)_2_.6H_2_O (116.6 mg, 0.4 mmol) was added into 1 ml dmf with thorough stir for 5 minutes. After filtration, the purple filtrate was carefully laid on the surface with a solution of NaN(CN)_2_ (89.1 mg, 1 mmol) in 1 ml dmf and 4 ml *i*-PrOH. Purple block crystals were obtained after five days.

### Refinement

H atoms were positioned geometrically and refined using a riding model, with C—H = 0.93–0.96 Å, and with *U*_iso_(H) = 1.5*U*_eq_(C) or 1.2*U*_eq_(C) for methyl and formyl H atoms, respectively.

### Crystal data

**Table:**

[Co(C_2_N_3_)_2_(C_3_H_7_NO)_2_]	*Z* = 1
*M**_r_* = 337.22	*F*(000) = 173
Triclinic, *P*1	*D*_x_ = 1.526 Mg m^−^^3^
Hall symbol: -P 1	Mo *K*α radiation, λ = 0.71073 Å
*a* = 6.4315 (13) Å	Cell parameters from 1585 reflections
*b* = 7.3879 (15) Å	θ = 4.5–29.1°
*c* = 8.6210 (17) Å	µ = 1.19 mm^−^^1^
α = 105.69 (3)°	*T* = 150 K
β = 107.94 (3)°	Block, purple
γ = 96.19 (3)°	0.22 × 0.18 × 0.15 mm
*V* = 366.93 (17) Å^3^	

### Data collection

**Table:**

Rigaku Saturn724+ diffractometer	1319 independent reflections
Radiation source: fine-focus sealed tube	1242 reflections with *I* > 2σ(*I*)
Graphite monochromator	*R*_int_ = 0.015
ω scans	θ_max_ = 25.3°, θ_min_ = 4.0°
Absorption correction: multi-scan (*CrystalClear*; Rigaku, 2008)	*h* = −7→7
*T*_min_ = 0.845, *T*_max_ = 1.000	*k* = −8→7
2514 measured reflections	*l* = −10→10

### Refinement

**Table:**

Refinement on *F*^2^	Primary atom site location: structure-invariant direct methods
Least-squares matrix: full	Secondary atom site location: difference Fourier map
*R*[*F*^2^ > 2σ(*F*^2^)] = 0.025	Hydrogen site location: inferred from neighbouring sites
*wR*(*F*^2^) = 0.061	H-atom parameters constrained
*S* = 1.04	*w* = 1/[σ^2^(*F*_o_^2^) + (0.0266*P*)^2^ + 0.119*P*] where *P* = (*F*_o_^2^ + 2*F*_c_^2^)/3
1319 reflections	(Δ/σ)_max_ < 0.001
97 parameters	Δρ_max_ = 0.22 e Å^−^^3^
0 restraints	Δρ_min_ = −0.24 e Å^−^^3^

### Special details

**Table:**

Geometry. All e.s.d.'s (except the e.s.d. in the dihedral angle between two l.s. planes) are estimated using the full covariance matrix. The cell e.s.d.'s are taken into account individually in the estimation of e.s.d.'s in distances, angles and torsion angles; correlations between e.s.d.'s in cell parameters are only used when they are defined by crystal symmetry. An approximate (isotropic) treatment of cell e.s.d.'s is used for estimating e.s.d.'s involving l.s. planes.
Refinement. Refinement of *F*^2^ against ALL reflections. The weighted *R*-factor *wR* and goodness of fit *S* are based on *F*^2^, conventional *R*-factors *R* are based on *F*, with *F* set to zero for negative *F*^2^. The threshold expression of *F*^2^ > σ(*F*^2^) is used only for calculating *R*-factors(gt) *etc*. and is not relevant to the choice of reflections for refinement. *R*-factors based on *F*^2^ are statistically about twice as large as those based on *F*, and *R*- factors based on ALL data will be even larger.

### Fractional atomic coordinates and isotropic or equivalent isotropic displacement parameters (Å^2^)

**Table:**

	*x*	*y*	*z*	*U*_iso_*/*U*_eq_	
Co1	0.5000	0.0000	0.5000	0.03475 (14)	
O1	0.7337 (2)	0.1252 (2)	0.75360 (17)	0.0458 (3)	
N1	0.3153 (3)	−0.1843 (2)	0.5846 (2)	0.0450 (4)	
N2	0.3184 (3)	0.2157 (2)	0.5512 (2)	0.0461 (4)	
N3	0.2307 (3)	−0.4729 (2)	0.6597 (2)	0.0540 (5)	
N4	1.0908 (3)	0.2096 (2)	0.9389 (2)	0.0429 (4)	
C1	0.2790 (3)	−0.3244 (3)	0.6133 (2)	0.0361 (4)	
C2	0.2822 (3)	0.3654 (3)	0.5966 (2)	0.0335 (4)	
C3	0.9359 (3)	0.1312 (3)	0.7855 (2)	0.0397 (4)	
H3C	0.9817	0.0763	0.6944	0.048*	
C4	1.0358 (5)	0.3018 (4)	1.0885 (3)	0.0620 (6)	
H4A	0.8777	0.2958	1.0544	0.093*	
H4B	1.1137	0.4337	1.1379	0.093*	
H4C	1.0796	0.2367	1.1720	0.093*	
C5	1.3232 (4)	0.2040 (4)	0.9659 (3)	0.0666 (7)	
H5A	1.3381	0.1399	0.8582	0.100*	
H5B	1.3753	0.1357	1.0456	0.100*	
H5C	1.4105	0.3329	1.0117	0.100*	

### Atomic displacement parameters (Å^2^)

**Table:**

	*U*^11^	*U*^22^	*U*^33^	*U*^12^	*U*^13^	*U*^23^
Co1	0.0364 (2)	0.02626 (19)	0.0418 (2)	0.00779 (14)	0.01291 (16)	0.01186 (15)
O1	0.0401 (8)	0.0490 (8)	0.0434 (8)	0.0089 (6)	0.0120 (7)	0.0100 (7)
N1	0.0503 (10)	0.0333 (9)	0.0546 (11)	0.0081 (7)	0.0214 (9)	0.0163 (8)
N2	0.0510 (10)	0.0356 (9)	0.0582 (11)	0.0160 (8)	0.0235 (9)	0.0177 (8)
N3	0.0846 (14)	0.0345 (9)	0.0629 (12)	0.0196 (9)	0.0489 (11)	0.0178 (9)
N4	0.0446 (10)	0.0461 (9)	0.0336 (9)	0.0087 (8)	0.0104 (8)	0.0102 (7)
C1	0.0374 (10)	0.0322 (10)	0.0381 (10)	0.0096 (8)	0.0155 (9)	0.0070 (8)
C2	0.0331 (9)	0.0331 (10)	0.0348 (10)	0.0050 (8)	0.0114 (8)	0.0127 (8)
C3	0.0451 (12)	0.0366 (10)	0.0370 (11)	0.0083 (9)	0.0145 (9)	0.0110 (8)
C4	0.0828 (18)	0.0611 (14)	0.0381 (12)	0.0177 (13)	0.0210 (12)	0.0087 (11)
C5	0.0450 (13)	0.0853 (18)	0.0556 (15)	0.0097 (12)	0.0047 (11)	0.0174 (13)

### Geometric parameters (Å, º)

**Table:**

Co1—N2^i^	2.1061 (17)	N4—C3	1.313 (3)
Co1—N2	2.1061 (17)	N4—C5	1.448 (3)
Co1—O1	2.1157 (17)	N4—C4	1.452 (3)
Co1—O1^i^	2.1157 (17)	C2—N3^iii^	1.295 (2)
Co1—N1	2.1254 (17)	C3—H3C	0.9300
Co1—N1^i^	2.1254 (17)	C4—H4A	0.9600
O1—C3	1.237 (2)	C4—H4B	0.9600
N1—C1	1.145 (2)	C4—H4C	0.9600
N2—C2	1.144 (2)	C5—H5A	0.9600
N3—C2^ii^	1.295 (2)	C5—H5B	0.9600
N3—C1	1.304 (2)	C5—H5C	0.9600
			
N2^i^—Co1—N2	180.00 (11)	C3—N4—C4	121.59 (19)
N2^i^—Co1—O1	90.92 (7)	C5—N4—C4	117.31 (19)
N2—Co1—O1	89.08 (7)	N1—C1—N3	173.5 (2)
N2^i^—Co1—O1^i^	89.08 (7)	N2—C2—N3^iii^	173.04 (19)
N2—Co1—O1^i^	90.92 (7)	O1—C3—N4	124.73 (18)
O1—Co1—O1^i^	180.0	O1—C3—H3C	117.6
N2^i^—Co1—N1	88.01 (7)	N4—C3—H3C	117.6
N2—Co1—N1	91.99 (7)	N4—C4—H4A	109.5
O1—Co1—N1	90.34 (7)	N4—C4—H4B	109.5
O1^i^—Co1—N1	89.66 (7)	H4A—C4—H4B	109.5
N2^i^—Co1—N1^i^	91.99 (7)	N4—C4—H4C	109.5
N2—Co1—N1^i^	88.01 (7)	H4A—C4—H4C	109.5
O1—Co1—N1^i^	89.66 (7)	H4B—C4—H4C	109.5
O1^i^—Co1—N1^i^	90.34 (7)	N4—C5—H5A	109.5
N1—Co1—N1^i^	180.00 (8)	N4—C5—H5B	109.5
C3—O1—Co1	121.36 (13)	H5A—C5—H5B	109.5
C1—N1—Co1	151.54 (16)	N4—C5—H5C	109.5
C2—N2—Co1	159.60 (16)	H5A—C5—H5C	109.5
C2^ii^—N3—C1	120.72 (16)	H5B—C5—H5C	109.5
C3—N4—C5	121.09 (18)		

Symmetry codes: (i) −*x*+1, −*y*, −*z*+1; (ii) *x*, *y*−1, *z*; (iii) *x*, *y*+1, *z*.
